# A mechanistic modeling approach to assessing the sensitivity of outcomes of water, sanitation, and hygiene interventions to local contexts and intervention factors

**DOI:** 10.1016/j.idm.2025.02.002

**Published:** 2025-02-03

**Authors:** Andrew F. Brouwer, Alicia N.M. Kraay, Mondal H. Zahid, Marisa C. Eisenberg, Matthew C. Freeman, Joseph N.S. Eisenberg

**Affiliations:** a Department of Epidemiology, University of Michigan, Michigan, USA; b Institute for Disease Modeling, a Program Within the Global Health Division of the Bill and Melinda Gates Foundation, Seattle, WA, USA; c Rollins School of Public Health, Emory University, Atlanta, GA, USA

**Keywords:** Water, Sanitation, Hygiene, Randomized controlled trial, Intervention, Disease transmission model, Simulation

## Abstract

Diarrheal disease is a leading cause of morbidity and mortality in young children. Water, sanitation, and hygiene (WASH) improvements have historically been responsible for major public health gains, but many individual interventions have failed to consistently reduce diarrheal disease burden. Analytical tools that can estimate the potential impacts of individual WASH improvements in specific contexts would support program managers and policymakers to set targets that would yield health gains. We developed a disease transmission model to simulate an intervention trial with a single intervention. We accounted for contextual factors, including preexisting WASH conditions and baseline disease prevalence, as well as intervention WASH factors, including community coverage, compliance, efficacy, and the intervenable fraction of transmission. We illustrated the sensitivity of intervention effectiveness to the contextual and intervention factors in each of two plausible disease transmission scenarios with the same disease transmission potential and intervention effectiveness but differing baseline disease burden and contextual/intervention factors. Whether disease elimination could be achieved through a single factor depended on the values of the other factors, so that changes that could achieve disease elimination in one scenario could be ineffective in the other scenario. Community coverage interacted strongly with both the contextual and the intervention factors. For example, the positive impact of increasing intervention community coverage increased non-linearly with increasing intervention compliance. With lower baseline disease prevalence in Scenario 1 (among other differences), our models predicted substantial reductions could be achieved with relatively low coverage. In contrast, in Scenario 2, where baseline disease prevalence was higher, high coverage and compliance were necessary to achieve strong intervention effectiveness. When developing interventions, it is important to account for both contextual conditions and the intervention parameters. Our mechanistic modeling approach can provide guidance for developing locally specific policy recommendations.

## 1 Introduction

Diarrheal disease is a leading cause of morbidity and mortality in young children, with an estimated 500,000 children under 5 years dying from diarrheal disease each year (Troeger et al., 2018; Vos et al., 2020; Wolf et al., 2023). Diarrheal disease is primarily caused by enteropathogens spread by fecal–oral pathways through contaminated environments, such as water, food, and fomites. Much of this burden is in low- and middle-income countries (LMICs) and among people living in poverty (Julian, 2016). Many enterpathogens are not good vaccine candidates, and those that are (e.g., rotavirus) can be hard to administer in the field (e.g., because of cold-chain requirements (Ashok et al., 2017)) or suffer from differential effectiveness (Jonesteller et al., 2017). Thus, preventive approaches for reducing enteric infections interventions are essential.

Historically, large-scale Water, Sanitation, and Hygiene (WASH) improvements have been responsible for major public health gains by greatly reducing exposure to fecal pathogens, demonstrating the potential effectiveness of WASH in reducing the burden (Contreras & Eisenberg, 2019; Harris & Helgertz, 2019). Yet many trialed interventions, especially in the most disadvantaged areas where enteric infections are endemic, have failed to consistently reduce the burden of diarrheal disease (Clasen et al., 2014; Knee et al., 2021; Luby et al., 2018; Rogawski McQuade et al., 2020; Null et al., 2018; Patil et al., 2015; Pickering et al., 2015). A recent meta-analysis of WASH intervention randomized controlled trials (RCTs) demonstrated that while WASH interventions can reduce diarrhea in children in low-resource settings overall (Wolf et al., 2022), the heterogeneity across the aggregated trials is substantial, with many of the more recent, large-scale trials finding less-than-expected or null results (Clasen et al., 2014; Knee et al., 2021; Luby et al., 2018; Rogawski McQuade et al., 2020; Null et al., 2018; Patil et al., 2015; Pickering et al., 2015).

Difficulties in achieving consistent reduction of diarrheal burden are caused by multiple factors. First, local contexts can vary widely in terms of preexisting WASH conditions (i.e., WASH infrastructure in place prior to the intervention) and disease prevalence, among other factors. These differences have made it difficult to apply the results from studies conducted in one location to other locations. Second, interventions are imperfect. For example, 1) they may not block transmission along all transmission pathways (e.g., a water chlorination intervention will not reduce disease from exposure to animal feces or contaminated food), 2) the intervention coverage within the target population may not be sufficient to confer indirect protection, 3) the intervention may provide access to improved WASH but not ensure compliance, or 4) the direct efficacy of the provided interventions on reducing transmission to the users may be limited (Cumming et al., 2019; Pickering et al., 2019). Other factors are important as well, such as bias and inconsistency in reporting diarrhea and differences in the pathogens and taxa responsible for diarrheal disease in different locations (Platts et al., 2015).

Analytical tools that can dynamically estimate the potential impacts of individual WASH improvements would support program managers and policymakers to set targets for investments to yield anticipated health gains. For example, with a given budget, should a program aim for greater compliance with an intervention or higher efficacy, if the goal is to reduce diarrheal burden? Mechanistic transmission models can be enhanced to help implementors design optimal intervention strategies by accounting for location-specific contextual factors such as disease prevalence and preexisting WASH conditions. One important strength of mechanistic approaches is their ability to generalize from available context-specific epidemiological findings to other contexts and counterfactual scenarios, and there is a need for tools that can generalize WASH trial results to other contexts.

Our objective was to develop a model to dynamically simulate diarrheal disease outcomes under various contextual and WASH intervention factors to understand which had the greatest impact on resulting disease burden. We previously developed a mechanistic model to simulate WASH trials and applied it to the WASH Benefits Bangladesh trial (Brouwer et al., 2022). Here, we aim to 1) demonstrate and estimate interactions between each of the contextual and intervention WASH factors and their impact on intervention efficacy and 2) increase the accessibility of the modeling framework for trialists and policymakers. This work will build our understanding of WASH interventions and improve the design of future trials.

## 2 Methods

**WASH factors.** In a typical single-intervention RCT, there are two populations enrolled, called arms: a control arm that does not receive the intervention and an intervention arm that does receive the intervention. Enrolled participants are randomly assigned to an arm, and effectiveness is defined as the percent reduction in diarrheal prevalence in the intervention arm compared to the control arm at the follow-up period (e.g., one year after interventions were implemented). For infectious disease RCTs, geographic clusters are often used to reduce the possibility of spillover of effects between participants. In this analysis, we explore how effectiveness of a single intervention depends on six contextual or intervention WASH factors.

• *Preexisting WASH conditions.* We account for the fraction of the population that already has WASH infrastructure comparable to that provided by the intervention.
• *Disease transmission potential.* We summarize disease transmission potential in the absence of intervention using the basic reproduction number $R_{0}$. Note that because the control-arm disease prevalence is determined by $R_{0}$ (given the values of other the other factors), we will not independently vary the control-arm disease prevalence in this analysis.
• *Intervention compliance.* We account for the fraction of participants assigned to an intervention that are actually using it. Compliance includes both fidelity (whether the intervention was delivered) and adherence (whether participants used the intervention).
• *Intervenable fraction of transmission.* Diarrheal disease pathogens are transmitted along multiple pathways, often summarized by an “F-diagram”: fluids, food, flies, fields, fauna, etc. Any individual intervention typically targets one or a few of these pathways, but not all of them, and each pathway is responsible for a different fraction of the total disease transmission potential. We account for how much transmission the intervention could prevent if it were perfectly efficacious and fully adopted. For trials that combine multiple interventions, the intervenable fraction can be thought of as the fraction of transmission that a combination of interventions could block.
• *Intervention efficacy.* Interventions do not perfectly prevent transmission along the pathways that they impact. We account for how much transmission (or shedding into the environment) the intervention prevents.
• *Community coverage.* In many trials, not everyone in the community is provided the intervention. We account for the fraction of the population that is enrolled in the trial.

Each of these factors is specifically accounted for in our transmission model, described below. Many of these factors can be measured for a given context. Preexisting WASH conditions could be determined from a local census and survey, if not already approximately known. Baseline disease potential is closely tied to baseline disease prevalence, and baseline disease prevalence is typically measured as part of intervention planning. Intervention efficacy, compliance, and community coverage are each intervention design parameters that would be specified or approximated prior to intervention implementation and may be able to be optimized based on the intervention chosen. The remaining factor, intervenable fraction of transmission, will generally be more difficult to directly measure and thus may be a primary target for sensitivity analysis. Because intervenable fraction likely varies by pathogen, intervenable fraction may be able to be estimated from local distribution of diarrhea-causing pathogens once pathogen-specific intervenable fractions are better understood.

## 3 Model

Our compartmental transmission model, denoted SISE-RCT, is a susceptible-infectious-susceptible (SIS) model with transmission through one or more environmental (E) compartments. To approximate the outcomes of a RCT, we solve for the model's steady state in an endemic setting (Brouwer et al., 2022). The SISE-RCT model accounts for the six mechanistic WASH factors outlined above that underlie WASH RCT results. In the case of a single intervention, the population is partitioned into the fraction with regular exposure (those not enrolled or included in the intervention and those not compliant), and the fraction with exposure or shedding attenuated by the intervention (those compliant with the intervention or an equivalent preexisting WASH condition). Susceptible and infectious individuals with regular exposure are designated $S_{-}$ and $I_{-}$, and those with exposure or shedding attenuated by the intervention are designated $S_{+}$ and $I_{+}$ (all human compartments are expressed as fractions of the overall population). The intervention and control arms are simulated separately, and both the regular and attenuated exposure populations are modeled in both arms, accounting for the fraction of population not enrolled in the study (ω, i.e., the *community coverage*), the fraction of the population with *preexisting WASH conditions* ($\rho_{0})$, and *intervention compliance* (ρ). Individuals with regular exposure are either in the study but not compliant to the intervention (ω(1−ρ)) or are not in the study and do not have preexisting WASH conditions ($\left(1-\omega \right)\left(1-\rho_{0}\right)$). Individuals with attenuated exposure are either in the study and compliant to the intervention (ωρ) or are not in the study but have preexisting WASH conditions ($\left(1-\omega \right)\rho_{0}$). Note that individuals who had preexisting WASH conditions and received the intervention are included in the ωρ term; we assume that trial never results in less protection, e.g., someone with preexisting WASH conditions does not move to the regular exposure group because of non-compliance with the intervention, since they would use their pre-existing WASH condition and retain attenuated exposure. Hence, the population fractions of the attenuated and regular exposure populations are given by

(1) $$\begin{array}{c} N_{+}=\omega \rho +\left(1-\omega \right)\rho_{0}, \end{array}$$

$\begin{array}{c} N_{-}=\omega \left(1-\rho \right)+\left(1-\omega \right)\left(1-\rho_{0}\right), \end{array}$ respectively.

Once infected, individuals clear the infection at rate γ, returning to the susceptible compartment. An environmental compartment is characterized by the shedding of pathogens by an infectious person into the environment (α), the decay of pathogens in the environment (ξ), and the transmission of pathogens from the environment to susceptible individuals (β). We assume that the ingestion of pathogens has a negligible effect on the environmental concentration of pathogens (Li et al., 2009). For the single-intervention model, the environment (modeled as a concentration) is partitioned into the environmental pathway that is affected by the intervention $E_{1}$, either in terms of shedding into or transmission from the environment, and the environmental pathway that is not affected by the intervention $E_{2}$, with the same subscripts on α, ξ, and β. For example, $E_{1}$ could be pathogens in water for an intervention that targets water, with $E_{2}$ representing all other potential transmission pathways (e.g., fomites, food, etc). The relative magnitude of shedding into $E_{1}$ and relative transmission from $E_{1}$ for the attenuated compared to the exposed populations are given by the *intervention efficacy* parameters $\phi_{\alpha_{1}}$ and $\phi_{\beta_{1}}$, respectively. That is, if $\beta_{1}$ is the transmission from $E_{1}$ without the intervention and $\bar{\beta_{1}}$ is the transmission from $E_{1}$ with the intervention, then $\phi_{\beta_{1}}=\bar{\beta_{1}}/\beta_{1}$, and $\phi_{\alpha_{1}}$ is defined analogously. Using intervention terminology, ϕ is 1 minus the efficacy of the intervention, e.g., ϕ would be 0.8 for a 20% efficacious intervention.

**Fig. 1:**
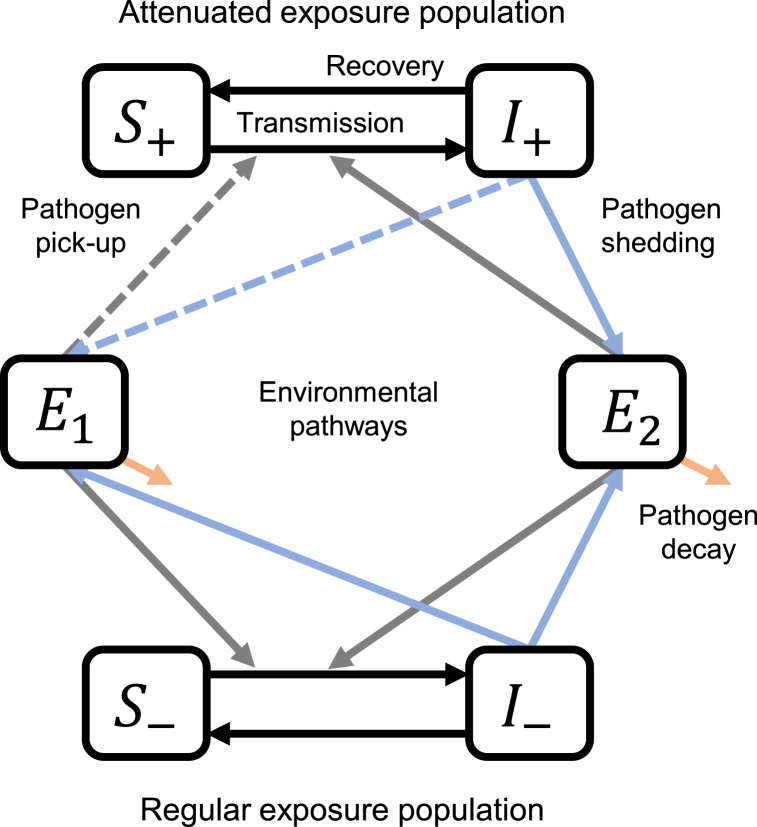
**Single-intervention SISE-RCT model diagram with an attenuated exposure population and a regular exposure population interacting through shared environments.** The SISE-RCT model is a compartmental susceptible-infectious-susceptible (SIS) model with transmission through environmental (E) compartments and simulated to steady state to approximate an RCT. The black lines denote infection and recovery, the blue lines denote shedding from infectious individuals into environmental compartments, the grey lines denote pick-up of pathogens from the environment by susceptible individuals, and the orange lines denote environmental pathogen decay. $S_{+}$ and $I_{+}$ denote susceptible and infectious fraction of the attenuate exposure population, and $S_{-}$ and $I_{-}$ denote susceptible and infectious fraction of the regular exposure population.

The SISE-RCT parameters are given in Table 1, and a model diagram is given in Fig. 1. The full equations are given below (Eq (2)). The two transmission terms $\beta_{1}E_{1}$ and $\beta_{2}E_{2}$ denote transmission from the environmental pathway attenuated by the intervention ($E_{1})$ and from the environmental pathway not attenuated by the intervention ($E_{2}$), respectively. The transmission term $\beta_{1}E_{1}$ is attenuated (through reduced contact or reduced susceptibility) by $\phi_{\beta_{1}}$ only for people in the attenuated exposure group ($S_{+})$, and contamination of that environmental pathway is attenuated (through sequestration or elimination of pathogens) by $\phi_{\alpha_{1}}$ only for infectious people of that same group ($I_{+})$. There is no attenuation of transmission to or shedding from the environmental pathway not affected by the intervention ($E_{2}$). Parameters ω, ρ, and $\rho_{0}$ do not appear in these equations but are accounted for in the constraints, as discussed below. For brevity, we omit the $\frac{dS}{dt}$ equations, each of which is given by $\frac{dS}{dt}=-\frac{dI}{dt}$ for the corresponding subpopulation.

(2) $$\begin{array}{c} \frac{dI_{+}}{dt}=\left(\phi_{\beta_{1}}\beta_{1}E_{1}+\beta_{2}E_{2}\right)S_{+}-\gamma I_{+}\text{,} \end{array}$$

$$\frac{dI_{-}}{dt}=\left(\beta_{1}E_{1}+\beta_{2}E_{2}\right)S_{-}-\gamma I_{-}\text{,}$$

$$\frac{dE_{1}}{dt}=\alpha_{1}\left(\phi_{\alpha_{1}}I_{+}+I_{-}\right)-\xi_{1}E_{1}\text{,}$$

$$\frac{dE_{2}}{dt}=\alpha_{2}\left(I_{+}+I_{-}\right)-\xi_{2}E_{2}\text{.}$$

**Table 1:** **Parameters of the SISE-RCT model in two scenarios.** The SISE-RCT model is a compartmental susceptible-infectious-susceptible (SIS) model with transmission through environmental (E) compartments and simulated to steady state to approximate an RCT. The baseline intervention effectiveness ($\varepsilon_{0}$) in both scenarios is 50%, but the WASH parameters and control-arm disease prevalence differ across scenarios.

			Scenario 1		Scenario 2	
Parameter	Definition	Sensitivity range	Baseline value	Disease elimination value	Baseline value	Disease elimination value
$\rho_{0}$	Preexisting WASH conditions (fraction of individuals in the community with intervention-level WASH infrastructure)	0–1	0.25	0.31	0	–
ρ	Compliance (fraction of individuals in intervention arm using intervention)	0–1	0.75	–	0.50	0.92
$R_{0}=R_{0,1}+R_{0,2}$	Transmission potential in the absence of intervention (basic reproduction number)	1–2	1.25	1.20	1.25	1.12
$\pi_{c}^{*}$	Control-arm disease prevalence	a	6.4%	2.8%	20.0%	10.7%
$\theta =R_{0,1}/R_{0}$	Intervenable fraction (fraction of transmission that the intervention could theoretically prevent	0–1	0.75	0.88	0.35	0.65
$1-\varphi_{\alpha_{1}}$	Intervention efficacy for reducing shedding	–	0	–	0	–
$1-\varphi_{\beta_{1}}$	Intervention efficacy for reducing transmission	0–1	0.75	0.98	0.83	–
ω	Community coverage fraction (fraction of community included in the intervention trial)	0–1	0.11	0.22	0.75	–

a Control-arm disease prevalence is a function of the transmission potential given the values of the other factors and was not varied independently of $R_{0}$.

To find the steady state values (denoted by ∗) for the human compartments in the intervention arm, we first set the derivatives of $E_{1}$ and $E_{2}$ to 0 and solve for those compartment's steady state values as a function of $I_{+}$ and $I_{-}$.

(3) $$\begin{array}{c} E_{1}^{*}=\alpha_{1}\left(\phi_{\alpha_{1}}I_{+}^{*}+I_{-}^{*}\right)/\xi_{1}, \end{array}$$

$$E_{2}^{*}=\alpha_{2}\left(I_{+}^{*}+I_{-}^{*}\right)/\xi_{2}\text{.}$$

Then, we set the derivatives of $I_{+}$ and $I_{-}$ to 0, simplify out the environmental compartments using Eqn (3), and divide both sides by γ.

(4) $$\begin{array}{c} 0=\left(\phi_{\beta_{1}}\frac{\beta_{1}\alpha_{1}}{\xi_{1}\gamma }\left(\phi_{\alpha_{1}}I_{+}^{*}+I_{-}^{*}\right)+\frac{\beta_{2}\alpha_{2}}{\xi_{2}\gamma }\left(I_{+}^{*}+I_{-}^{*}\right)\right)S_{+}^{*}-I_{+}^{*},\\ 0=\left(\frac{\beta_{1}\alpha_{1}}{\xi_{1}\gamma }\left(\phi_{\alpha_{1}}I_{+}^{*}+I_{-}^{*}\right)+\frac{\beta_{2}\alpha_{2}}{\xi_{2}\gamma }\left(I_{+}^{*}+I_{-}^{*}\right)\right)S_{-}^{*}-I_{-}^{*}\text{.} \end{array}$$

In these equations, parameters $\beta_{i}$, $\alpha_{i}$, $\xi_{i}$, and γ cannot be separately identified from the values of the steady states, and so we define the identifiable parameter combinations R0,i:=βiαiξiγ. Then, Eq (4) simplify to the following.

(5) $$\begin{array}{c} 0=\left(\phi_{\beta_{1}}R_{0,1}\left(\phi_{\alpha_{1}}I_{+}^{*}+I_{-}^{*}\right)+R_{0,2}\left(I_{+}^{*}+I_{-}^{*}\right)\right)S_{+}^{*}-I_{+}^{*},\\ 0=\left(R_{0,1}\left(\phi_{\alpha_{1}}I_{+}^{*}+I_{-}^{*}\right)+R_{0,2}\left(I_{+}^{*}+I_{-}^{*}\right)\right)S_{-}^{*}-I_{-}^{*}\text{.} \end{array}$$

We call the $R_{0,i}$ the *pathway-specific reproduction numbers* for transmission through each environment $E_{i}$, as they are the basic reproduction numbers for the system with only that transmission pathway and with no intervention (as shown in the supplementary material). For this specific model, the overall basic reproduction number in the absence of intervention (i.e., with $\phi_{\beta_{1}}=\phi_{\alpha_{1}}=1$) is $R_{0}=R_{0,1}+R_{0,2}$, denoting the sum of the transmission potential through the pathway that would be affected by the intervention ($R_{0,1}$) and the pathway that would not be affected by the intervention ($R_{0,2}$). The *intervenable fraction of transmission* (based on the strength of the transmission pathway targeted by the specific intervention) is $\theta =R_{0,1}/R_{0}$. In this paper, $R_{0}$ will denote the *disease transmission potential* in the absence of intervention.

To get the steady states solutions for our four state variables ($S_{+}^{*}$, $I_{+}^{*}$, $S_{-}^{*}$, $I_{-}^{*}$), we solve the nonlinear system of equations (Eq (3)) subject to the constraints $S_{+}^{*}+I_{+}^{*}=N_{+}$ and $S_{-}^{*}+I_{-}^{*}=N_{-}$, where $N_{+}$ and $N_{-}$ are given in Eq (1). We numerically solved this system using the nleqslv package in R. This approach is more computationally efficient than the differential equation simulation approach we used previously (Brouwer et al., 2022). We solve for the steady state in the control arm with the same parameters as the intervention arm except that $\rho =\rho_{0}$.

The prevalence of disease in the population is denoted $\pi^{*}=I_{+}^{*}+I_{-}^{*}$. The prevalence in the intervention arm $\pi^{*}$ is compared to the prevalence $\pi_{c}^{*}$ in the control arm. Then, intervention effectiveness (the RCT outcome) is defined as $\varepsilon =\left(\pi_{c}^{*}-\pi^{*}\right)/\pi_{c}^{*}$, namely the fractional reduction in prevalence in the intervention arm relative to the control arm. We say that the disease is eliminated if $\pi^{*}=0$, so that ε=100%.

We investigated the sensitivity of the intervention effectiveness to each WASH factor for each of two scenarios with different sets of parameters. We focused on local sensitivity rather than global sensitivity because intervention effectiveness will always be specific to a given context and planned intervention. We first solved for the steady state solution for each scenario as listed in Table 1. Scenario 1 is characterized by a greater fraction of preexisting WASH conditions, compliance, and intervenable fraction, while Scenario 2 is characterized by a greater intervention efficacy and community coverage. The specific parameter values of the WASH contextual and intervention factors were chosen to be largely different between the two scenarios but to result in a baseline intervention effectiveness $\varepsilon_{0}$ of 50% in both. The transmission potential was the same in both scenarios but the resulting control arm disease prevalence in Scenario 1 (6.4%) was much lower than that of Scenario 2 (20.0%) because of the differences in the other factors (particularly the preexisting WASH conditions). The scenarios were chosen to demonstrate how sensitivity to the WASH factors might be different in different plausible scenarios and are not intended to be representative of any specific intervention trial nor to represent the full breadth of plausible scenarios.

We varied each factor one at a time across the range of values given in Table 1, calculating the value needed to achieve disease elimination in that scenario. We also varied each pair of factors together (e.g., varying coverage and compliance together) to investigate potential interactions between factors. Only simulations with $\rho >\rho_{0}$ and $\pi_{c}^{*}>0$ were included to avoid simulation of situations where the intervention reduced use of WASH or in which an intervention was applied to a system with no disease. This model has been made publicly available as a web app (https://umich-biostatistics.shinyapps.io/sise_rct/), and the code is available in a Zenodo repository (https://doi.org/10.5281/zenodo.10950560) and in the supplementary material.

Note that the contextual factors, i.e., the preexisting WASH conditions and the transmission potential, are not modifiable in a real-world setting. In this analysis, changing these parameters represents the changing the location of the hypothetical trial and can help to reveal how the findings of a trial might generalize to other locations. While the sensitivity of intervention effectiveness to these parameters may be less relevant for trial planning in a specific location, it is important for developing a better understanding of the heterogeneity between trials and may also help to identify contexts where certain intervention approaches may be more effective than others.

## 4 Results

The intervention effectiveness outcome $\varepsilon_{0}$ in the Scenario 1 baseline, given by the parameters in Table 1, was 50%, with a steady-state prevalence of 6.4% in the control arm and 3.2% in the intervention arm. Disease elimination would have been achieved in this hypothetical intervention if any of the following conditions were met: 1) we increased the preexisting conditions so that 31% rather than 25% of the population already had comparable WASH infrastructure; 2) we reduced the disease potential transmission potential from $R_{0}$ = 1.25 to $R_{0}$ = 1.20, which is equivalent here to reducing the control-arm disease prevalence from 6.4% to 2.8%; 3) we increased the intervenable fraction of transmission from 75% to 88% (since there is an unknown, “true” value of the intervenable fraction, it may be more intuitive to think of this change as adding interventions until they target pathways responsible for 88% of the transmission potential); 4) we increased the efficacy of the intervention at reducing transmission from 75% to 88%; or 5) we increased the community coverage from 11% to 22%. Disease could not be eliminated by increasing intervention compliance from 75%, even to 100%.

The intervention effectiveness outcome $\varepsilon_{0}$ in the Scenario 2 baseline, given by the parameters in Table 1, was also 50%, with a steady-state prevalence of 20.0% in the control arm and 10.0% in the intervention arm. Disease elimination would have been achieved in this hypothetical intervention if 1) we increased intervention compliance from 50% to 92%; 2) we reduced the disease potential transmission potential from $R_{0}$ = 1.25 to $R_{0}$ = 1.12, which is equivalent to reducing the control-arm disease prevalence from 20.0% to 10.7%; or 3) we increased the intervenable fraction of transmission from 35% to 65%. The disease could not be eliminated with higher preexisting WASH conditions, higher efficacy, or higher community coverage.

**Fig. 2:**
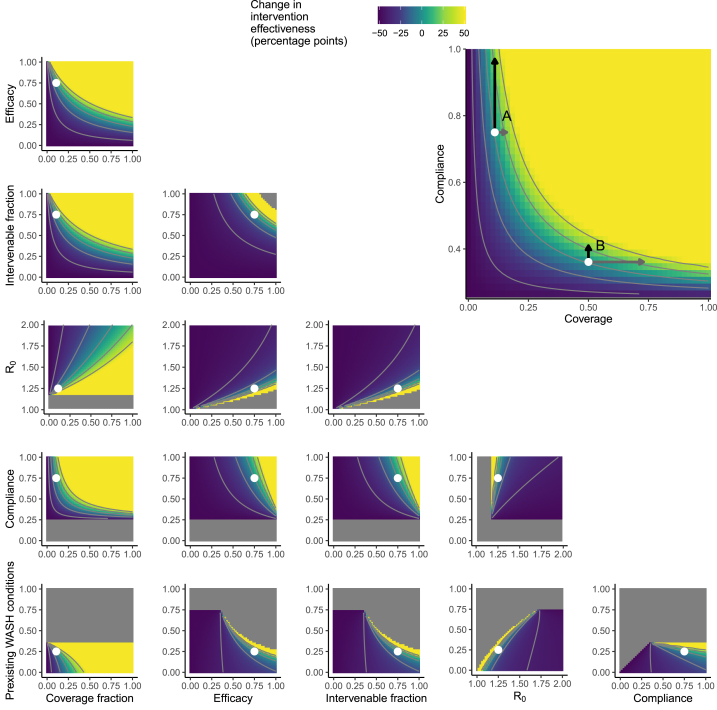
**Change in intervention effectiveness as a function of WASH intervention factors in Scenario 1.** The SISE-RCT model is a compartmental susceptible-infectious-susceptible (SIS) model with transmission through environmental (E) compartments and simulated to steady state to approximate an RCT. A single-intervention implementation of the model was simulated at the Scenario 1 baseline values given in Table 1 (indicated by the white points), and the heatmaps denote how intervention effectiveness changes depending on each pair of WASH factors. The six WASH factors are preexisting WASH conditions (fraction of individuals not enrolled in the intervention arm that are using preexisting infrastructure comparable to the intervention), compliance (fraction of individuals enrolled in the intervention arm that are using the intervention), disease transmission potential (summarize by the basic reproduction number $R_{0}$), intervenable fraction of transmission (how much of the transmission could be prevented in a perfect intervention), intervention efficacy (fraction reduction in transmission or shedding when using the intervention), and the community coverage fraction (fraction of the population enrolled in the trial). The inset enlarges the compliance vs coverage plot and overlays contour lines to show the interaction between the two factors on the change in intervention effectiveness. When coverage is low and compliance is high, it is easier to increase intervention effectiveness by increasing coverage, but when coverage is higher and compliance is low, then it is easier to increase intervention effectiveness by increasing compliance. The grey areas indicate inadmissible scenarios, i.e., where the intervention reduced WASH coverage ($\rho \leq \rho_{0})$ or disease prevalence was 0 in the control arm ($\pi_{c}^{*}=0$). Contours are given at multiples of 20 percentage points. WASH = water, sanitation, & hygiene.

**Fig. 3:**
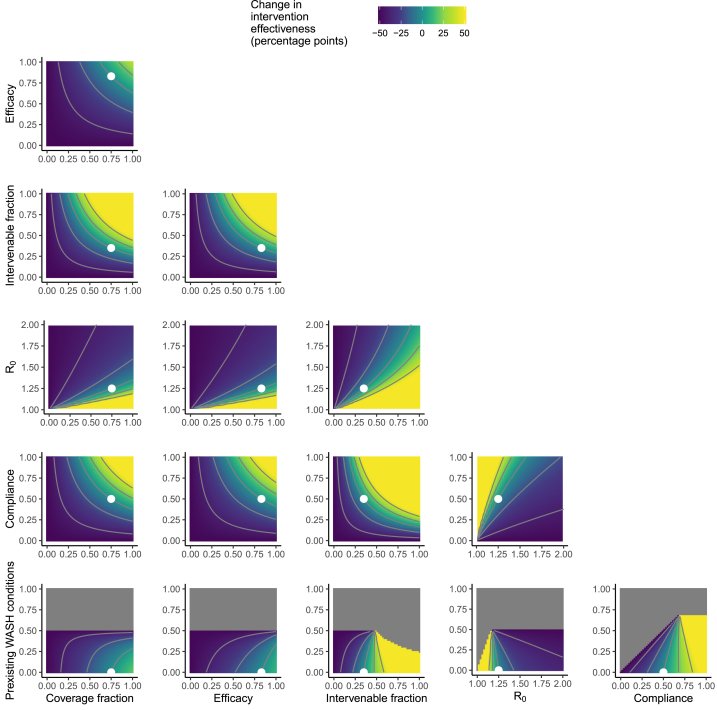
**Change in intervention effectiveness as a function of WASH intervention factors in Scenario 2.** Analogously to Fig. 2, a single-intervention implementation of the model was simulated at the Scenario 2 baseline values given in Table 1 (and indicated by the white points), and the heatmaps denote how intervention effectiveness changes depending on each pair of WASH factors. See Fig. 2 caption for additional information.

The change in intervention effectiveness ($\varepsilon -\varepsilon_{0})$ in percentage points as a function of each pair of the six parameters is given in Fig. 2 for Scenario 1 and in Fig. 3 for Scenario 2, with each scenario's baseline indicated by the white points. For many pairs of parameters, there was little evidence of an interaction between the factors (i.e., the contours of the heatmaps are approximately linear and parallel, except at extreme values). The primary exception to this pattern was coverage. In the inset in Fig. 2, we show, as an illustration, the interaction between coverage and compliance on the intervention effectiveness. When coverage is low and compliance is high (point A), it is easier to increase intervention effectiveness by increasing coverage (grey arrow, moving along the x-axis), but when coverage is higher and compliance is low (point B), then it is easier to increase intervention effectiveness by increasing compliance (black arrow, moving along the y-axis). “Easier” here does not reflect cost or feasibility but only the result of a unit change for each individual parameter. Cost-effectiveness is outside of the scope of this work but could be explored in future analysis. Similarly, the coverage needed to achieve disease elimination depended non-linearly on each of the other factors.

Increasing the fraction of the population with preexisting WASH conditions improved intervention effectiveness in Scenario 1 (Fig. 2) but decreased intervention effectiveness in Scenario 2 (Fig. 3). Increasing the fraction of the population with preexisting WASH conditions decreased prevalence in both the control and intervention arms, regardless of the specific scenario, but the *relative* reduction depended on the other WASH factors. In Scenario 2, for example, if the intervenable fraction were above 0.5 or if intervention compliance were above 0.75, then increasing baseline WASH conditions would result in increased intervention effectiveness.

The reader can explore the sensitivity of the model for other values of the WASH factors on the web app available via https://umich-biostatistics.shinyapps.io/sise_rct/or using the code included as supplementary material.

## 5 Discussion

We examined how the effectiveness of hypothetical single-intervention WASH RCTs depended on both contextual factors (baseline disease prevalence and preexisting WASH conditions) and intervention factors (community coverage, compliance, efficacy, and the intervenable fraction of transmission). Perhaps not surprisingly, the impact of changing one of these parameters was often highly dependent on the others. The effect of increasing community coverage, in particular, had a strong interaction with the other factors. For example, increasing the community coverage fraction could quickly lead to disease elimination if intervention compliance and efficacy were high, but have little impact if either were low. Our work demonstrates that it is important to understand the local, contextual conditions when developing relative priorities for an intervention. Our mechanistic modeling approach could allow for a tailored approach to designing interventions and WASH programs based on local conditions. For example, in some contexts with low baseline disease prevalence (like Scenario 1), substantial impacts might be achievable even with relatively low coverage. In contrast, in some contexts with high baseline disease prevalence (like Scenario 2), high coverage and compliance may be necessary to achieve strong intervention effectiveness. Although our work used disease elimination as an outcome, it could be similarly used to estimate how to achieve specific reduction targets short of elimination, which may be more realistic in many settings.

Our findings offer a potential explanation for the high heterogeneity in the results of WASH intervention studies (Wolf et al., 2022) as well as the less-than-expected effectiveness of interventions in recent, large WASH intervention trials (Clasen et al., 2014; Knee et al., 2021; Luby et al., 2018; Rogawski McQuade et al., 2020; Null et al., 2018; Patil et al., 2015; Pickering et al., 2015). An intervention that is effective in one location may be less effective in another location because of differences in the preexisting WASH infrastructure (e.g., the new location has unimproved latrines rather than open defecation) or differences in the disease pressure and prevalence.

Additionally, there may be substantial differences in the distribution of enteropathogens in locations, as demonstrated by the MAL-ED and GEMS studies (Kotloff et al., 2013; Liu et al., 2016; Platts et al., 2015). These pathogens may use different transmission pathways, and, as a result, the intervenable transmission fraction for the intervention may be different in different locations (Julian, 2016). For example, norovirus is one of the hardest pathogens to control, as it can exploit multiple transmission pathways. Norovirus was particularly important as a cause of diarrheal disease at the MAL-ED study site in Nepal but was not detected at the site in India (Platts et al., 2015). Thus, an intervention blocking only one pathway might be less likely to reduce overall disease prevalence at the Nepal site, compared with India site. Moreover, the intervenable fraction may vary temporally within a site, as the dominant diarrheal pathogens may vary seasonally in their incidence. Continuing with the norovirus example, single-intervention effectiveness might vary throughout the year and be less pronounced during cooler and wetter seasons, when norovirus is typically more prevalent (Ahmed et al., 2013).

The strength of this analysis lies in the mechanistic framework that allows us to connect diarrheal disease outcomes in a WASH intervention context to the specific, measurable WASH factors that characterize the location and the intervention. Because we were interested in providing a basic understanding of the drivers of successful interventions, we decided to use hypothetical WASH factor values that were plausible but not specific to an existing trial. However, this abstraction is also a limitation, as the real-world situations in randomized controlled trials will violate model assumptions, for example with spatial heterogeneity and clustering of compliance, baseline conditions, or infection prevalence. In using this model with real trial data, as we have done previously (Brouwer et al., 2022; Brouwer et al., 2024.), one approach is to apply the model to discrete geographic units (e.g., trial clusters) parameterized by local information rather than apply it to aggregated trial arms.

In the wake of the less-effective-than-expected large WASH intervention trial, a consensus group of WASH researchers called for a “pause for reflection” to re-evaluation the existing body of evidence (Cumming et al., 2019). A recent meta-analysis has suggested that WASH is effective at reducing diarrheal disease, though the outcomes are highly heterogeneous (Wolf et al., 2022). Our mechanistic modeling framework is another approach that is well-suited to re-evaluating existing evidence and generating hypotheses for causal explanations of the results of these trials. Ultimately, our work will help to provide evidence for developing locally specific policy recommendations and programmatic targets and for designing the next-generation WASH interventions (Amebelu et al., 2021; Levy & Eisenberg, 2019; Pickering et al., 2019).

## CRediT authorship contribution statement

**Andrew F. Brouwer:** Writing – review & editing, Writing – original draft, Visualization, Software, Methodology, Formal analysis, Conceptualization. **Alicia N.M. Kraay:** Writing – review & editing, Validation. **Mondal H. Zahid:** Writing – review & editing, Validation. **Marisa C. Eisenberg:** Writing – review & editing, Methodology. **Matthew C. Freeman:** Writing – review & editing, Funding acquisition, Conceptualization. **Joseph N.S. Eisenberg:** Writing – review & editing, Methodology, Funding acquisition, Conceptualization.

## Data availability statement

No data are associated with this article. The code is included as supplemental material and in the Zenodo repository at https://doi.org/10.5281/zenodo.10950560. The SISE-RCT web app with the single-intervention model is available at https://umich-biostatistics.shinyapps.io/sise_rct/.

## Declaration of competing interest

The authors declare the following financial interests/personal relationships which may be considered as potential competing interests: Joseph NS Eisenberg reports financial support was provided by Bill & Melinda Gates Foundation. Marisa C Eisenberg reports financial support was provided by National Science Foundation. Joseph NS Eisenberg reports a relationship with Bill & Melinda Gates Foundation that includes: travel reimbursement. Andrew F Brouwer reports a relationship with Bill & Melinda Gates Foundation that includes: travel reimbursement. Alicia NM Kraay reports a relationship with Bill & Melinda Gates Foundation that includes: employment. If there are other authors, they declare that they have no known competing financial interests or personal relationships that could have appeared to influence the work reported in this paper.

## References

[bib1] Ahmed S.M., Lopman B.A., Levy K. (2013). A systematic review and meta-analysis of the global seasonality of Norovirus. PLoS ONE.

[bib2] Amebelu A., Ban R., Bhagwan J., Brown J., Chilengi R., Chandler C., Colford J.M., Cumming O., Curtis V., Evans B.E., Freeman M.C., Guiteras R., Howard G., Humphrey J., Kang G., Kulabako R., Lanata C.F., Montgomery M.A., Pickering A.J., Wolf, J. (2021). The Lancet Commission on water, sanitation and hygiene, and health. Lancet.

[bib3] Ashok A., Brison M., LeTallec Y. (2017). Improving cold chain systems: Challenges and solutions. Vaccine.

[bib4] Brouwer A.F., Eisenberg M.C., Bakker K.M., Boerger S.N., Zahid M.H., Freeman M.C., Eisenberg J.N.S. (2022). Leveraging infectious disease models to interpret randomized controlled trials: controlling enteric pathogen transmission through water, sanitation, and hygiene interventions. PLOS Computational Biology.

[bib5] Brouwer AF, Zahid MH, Eisenberg MC, Arnold B.F., Ashraf S., Benjamin-Chung J., Colford J.M., Ercumen A., Luby S.P., Pickering A.J., Rahman M., Kraay A.N.M., Eisenberg J.N.S., Freeman M.C. (2024). Understanding the effectiveness of water, sanitation, and hygiene interventions: A counterfactual simulation approach to generalizing the outcomes of intervention trials. Environmental Health Perspectives.

[bib6] Clasen T., Boisson S., Routray P., Torondel B., Bell M., Cumming O., Ensink J., Freeman M., Jenkins M., Odagiri M., Ray S., Sinha A., Suar M., Schmidt W.-P. P. (2014). Effectiveness of a rural sanitation programme on diarrhoea, soil-transmitted helminth infection, and child malnutrition in odisha, India: A cluster-randomised trial. Lancet Glob Heal.

[bib7] Contreras J.D., Eisenberg J.N.S. (2019). Does basic sanitation prevent diarrhea? Contextualizing recent intervention trials through a historical lens. Int J Environ Res Public Health.

[bib8] Cumming O., Arnold B.F., Ban R., Clasen T., Esteves Mills J., Freeman M.C., Gordon B., Guiteras R., Howard G., Hunter P.R., Johnston R.B., Pickering A.J., Prendergast A.J., Prüss-Ustün A., Rosenboom J.W., Spears D., Sundberg S., Wolf J., Null C., Colford J.M (2019). The implications of three major new trials for the effect of water, sanitation and hygiene on childhood diarrhea and stunting: A consensus statement. BMC Medicine.

[bib9] Harris B., Helgertz J. (2019). Urban sanitation and the decline of mortality. The History of the Family.

[bib10] Jonesteller C.L., Burnett E., Yen C., Tate J.E., Parashar U.D. (2017). Effectiveness of rotavirus vaccination: A systematic review of the first decade of global postlicensure data, 2006–2016. Clinical Infectious Diseases.

[bib11] Julian T.R. (2016). Environmental transmission of diarrheal pathogens in low and middle income countries. Environ Sci Process Impacts.

[bib12] Knee J., Sumner T., Adriano Z., Anderson C., Bush F., Capone D., Casmo V., Holcomb D., Kolsky P., MacDougall A., Molotkova E., Braga J.M., Russo C., Schmidt W.P., Stewart J., Zambrana W., Zuin V., Nalá R., Cumming O., Brown J. (2021). Effects of an urban sanitation intervention on childhood enteric infection and diarrhea in maputo, Mozambique: A controlled before-and-after trial. Elife.

[bib13] Kotloff K.L., Nataro J.P., Blackwelder W.C., Nasrin D., Farag T.H., Panchalingam S., Wu Y., Sow S.O., Sur D., Breiman R.F., Faruque A.S.G., Zaidi A.K.M., Saha D., Alonso P.L., Tamboura B., Sanogo D., Onwuchekwa U., Manna B., Ramamurthy T., Levine M.M. (2013). Burden and aetiology of diarrhoeal disease in infants and young children in developing countries (the global enteric multicenter study, GEMS): A prospective, case-control study. Lancet.

[bib14] Levy K., Eisenberg J.N.S. (2019). Moving towards transformational WASH. Lancet Glob Heal.

[bib15] Li S., Eisenberg J.N.S., Spicknall I.H., Koopman J.S. (2009). Dynamics and control of infections transmitted from person to person through the environment. American Journal of Epidemiology.

[bib16] Liu J., Platts-Mills J.A., Juma J., Kabir F., Nkeze J., Okoi C., Operario D.J., Uddin J., Ahmed S., Alonso P.L., Antonio M., Becker S.M., Blackwelder W.C., Breiman R.F., Faruque A.S.G., Fields B., Gratz J., Haque R., Hossain A., Houpt E.R. (2016). Use of quantitative molecular diagnostic methods to identify causes of diarrhoea in children: A reanalysis of the GEMS case-control study. Lancet.

[bib17] Luby S.P., Rahman M., Arnold B.F., Unicomb L., Ashraf S., Winch P.J., Stewart C.P., Begum F., Hussain F., Benjamin-Chung J., Leontsini E., Naser A.M., Parvez S.M., Hubbard A.E., Lin A., Nizame F.A., Jannat K., Ercumen A., Ram P.K., Colford J.M. (2018). Effects of water quality, sanitation, handwashing, and nutritional interventions on diarrhoea and child growth in rural Bangladesh: A cluster randomised controlled trial. Lancet Glob Heal.

[bib19] Null C., Stewart C.P., Pickering A.J., Dentz H.N., Arnold B.F., Arnold C.D., Benjamin-Chung J., Clasen T., Dewey K.G., Fernald L.C.H., Hubbard A.E., Kariger P., Lin A., Luby S.P., Mertens A., Njenga S.M., Nyambane G., Ram P.K., Colford J.M. (2018). Effects of water quality, sanitation, handwashing, and nutritional interventions on diarrhoea and child growth in rural Kenya: A cluster-randomised controlled trial. Lancet Glob Heal.

[bib20] Patil S.R., Arnold B.F., Salvatore A.L., Briceno B., Ganguly S., Colford J.M., Gertler P.J. (2015). The effect of India’s total sanitation campaign on defecation behaviors and child health in rural Madhya Pradesh: A cluster randomized controlled trial. PLoS Medicine.

[bib21] Pickering A.J., Djebbari H., Lopez C., Coulibaly M., Alzua M.L. (2015). Effect of a community-led sanitation intervention on child diarrhoea and child growth in rural Mali: A cluster-randomised controlled trial. Lancet Glob Heal.

[bib22] Pickering A.J., Null C., Winch P.J., Mangwadu G., Arnold B.F., Prendergast A.J., Njenga S.M., Rahman M., Ntozini R., Benjamin-Chung J., Stewart C.P., Huda T.M.N., Moulton L.H., Colford J.M., Luby S.P., Humphrey J.H. (2019). The WASH Benefits and SHINE trials: Interpretation of WASH intervention effects on linear growth and diarrhoea. Lancet Glob Heal.

[bib23] Platts-Mills J.A., Babji S., Bodhidatta L., Gratz J., Haque R., Havt A., McCormick B.J.J., McGrath M., Olortegui M.P., Samie A., Shakoor S., Mondal D., Lima I.F.N., Hariraju D., Rayamajhi B.B., Qureshi S., Kabir F., Yori P.P., Mufamadi, B., Svensen E. (2015). Pathogen-specific burdens of community diarrhoea in developing countries: A multisite birth cohort study (MAL-ED). Lancet Glob Heal.

[bib18] Rogawski McQuade E.T., Platts-Mills J.A., Gratz J., Zhang J., Moulton L.H., Mutasa K., Majo F.D., Tavengwa N., Ntozini R., Prendergast A.J., Humphrey J.H., Liu J., Houpt E.R. (2020). Impact of water quality, sanitation, handwashing, and nutritional interventions on enteric infections in rural Zimbabwe: The sanitation hygiene infant nutrition efficacy (SHINE) trial. The Journal of Infectious Diseases.

[bib24] Troeger C., Blacker B., Khalil I.A., Rao P.C., Cao J., Zimsen S.R.M., Albertson S.B., Deshpande A., Farag T., Abebe Z., Adetifa I.M.O., Adhikari T.B., Akibu M., Al Lami F.H., Al-Eyadhy A., Alvis-Guzman N., Amare A.T., Amoako Y.A., Antonio C.A.T., Reiner R.C. (2018). Estimates of the global, regional, and national morbidity, mortality, and aetiologies of lower respiratory infections in 195 countries, 1990–2016: A systematic analysis for the global burden of disease study 2016. The Lancet Infectious Diseases.

[bib25] Vos, T., Lim, S.S., Abbafati, C., Abbas, K. M., Abbasi, M., Abbasifard, M., Abbasi-Kangevari, M., Abbastabar, H., Abd-Allah, F., Abdelalim, A., Abdollahi, M., Abdollahpour, I., Abolhassani, H., Aboyans, V., Abrams, E. M., Abreu, L. G., Abrigo, M. R. M., Abu-Raddad, L. J., Abushouk, A. I., ... Murray, C. J. L. (2020). Global burden of 369 diseases and injuries in 204 countries and territories, 1990–2019: A systematic analysis for the global burden of disease study 2019. Lancet, 396(10258), 1204–1222. 10.1016/S0140-6736(20)30925-9. PMC7567026 33069326

[bib26] Wolf J., Hubbard S., Brauer M., Ambelu A., Arnold B.F., Bain R., Bauza V., Brown J., Caruso B.A., Clasen T., Colford J.M., Freeman M.C., Gordon B., Johnston R.B., Mertens A., Prüss-Ustün A., Ross I., Stanaway J., Zhao J.T., Boisson, S. (2022). Effectiveness of interventions to improve drinking water, sanitation, and handwashing with soap on risk of diarrhoeal disease in children in low-income and middle-income settings: A systematic review and meta-analysis. Lancet.

[bib27] Wolf J., Johnston R.B., Ambelu A., Arnold B.F., Bain R., Brauer M., Brown J., Caruso B.A., Clasen T., Colford J.M., Mills J.E., Evans B., Freeman M.C., Gordon B., Kang G., Lanata C.F., Medlicott K.O., Prüss-Ustün A., Troeger C., Cumming O. (2023). Burden of disease attributable to unsafe drinking water, sanitation, and hygiene in domestic settings: A global analysis for selected adverse health outcomes. Lancet.

