# Supplementary material for: A mechanistic modeling approach to assessing the sensitivity of outcomes of water, sanitation, and hygiene interventions to local contexts and intervention factors
Source: Infect Dis Model. 2025 Feb 3;10(2):649–59. doi: 10.1016/j.idm.2025.02.002 (PMC11870245; doi:10.1016/j.idm.2025.02.002)
Supplement: Multimedia component 1 [file mmc1.docx]

**Supplementary material**

**Basic reproduction number**

Here we derive the basic reproduction number of the single-intervention SISE-RCT model with intervention $R_{0}^{\phi}$ and in the absence of intervention $R_{0}$, as well as justify the definitions of the pathway-specific reproduction numbers defined in the main text. We derive the basic reproduction for the Eq (2) in the main text using the Next Generation Matrix approach (developed by Diekmann et al. (1990), further explored by Van den Driessche and Watmough (2002)). We will not discuss the technical details of the Next Generation Matrix approach here and refer the reader to the previous citations and the introduction and overview of the topic by Brouwer (2022).

The single intervention SISE-RCT model (Eq (2)) has four infected compartments: $I_{+}$, $I_{-}$, $E_{1}$, and $E_{2}$, which are defined in the main text. We decompose these equations into vectors representing new infections and the terms denoting compartmental transfer.

$$\left[ \begin{matrix} \frac{dI_{+}}{dt} \\ \frac{dI_{-}}{dt} \\ \frac{dE_{1}}{dt} \\ \frac{dE_{2}}{dt} \end{matrix} \right]=\left[ \begin{matrix} {(\phi}_{\beta_{1}}\beta_{1}E_{1}+\beta_{2}E_{2})S_{+} \\ \left( \beta_{1}E_{1}+\beta_{2}E_{2} \right)S_{-} \\ 0 \\ 0 \end{matrix} \right]- \left[ \begin{matrix} \gamma I_{+} \\ \gamma I_{-} \\ \xi_{1}E_{1}-{\alpha_{1}(\phi}_{\alpha_{1}}I_{+}+I_{-}) \\ \xi_{2}E_{2}-\alpha_{2}\left( I_{+}+I_{-} \right) \end{matrix} \right]$$

We evaluate the Jacobian of these two vectors at the disease-free equilibrium. For simplicity, denote the fraction of the population with regular exposure as $\pi=N_{-}/(N_{+}+N_{-})$ and the fraction with attenuated exposure as $(1-\pi)$. Note that $\pi$ is defined by $\omega$, $\rho$, and $\rho_{0}$ through the relationships in Eq (1). The Jacobians F and V are as follows:

$$F=\left[ \begin{matrix} 0 & 0 & \phi_{\beta_{1}}\beta_{1}S_{+} & \beta_{2}S_{+} \\ 0 & 0 & \beta_{1}S_{-} & \beta_{2}S_{-} \\ 0 & 0 & 0 & 0 \\ 0 & 0 & 0 & 0 \end{matrix} \right]_{\text{DFE}}=\left[ \begin{matrix} 0 & 0 & \phi_{\beta_{1}}\beta_{1}(1-\pi) & \beta_{2}(1-\pi) \\ 0 & 0 & \beta_{1}\pi& \beta_{2}\pi\\ 0 & 0 & 0 & 0 \\ 0 & 0 & 0 & 0 \end{matrix} \right]$$

$$V=\left[ \begin{matrix} \gamma& 0 & 0 & 0 \\ 0 & \gamma& 0 & 0 \\ {{-\alpha}_{1}\phi}_{\alpha_{1}} & {-\alpha}_{1} & \xi_{1} & 0 \\ {-\alpha}_{2} & {-\alpha}_{2} & 0 & \xi_{2} \end{matrix} \right]_{\text{DFE}}=\left[ \begin{matrix} \gamma& 0 & 0 & 0 \\ 0 & \gamma& 0 & 0 \\ {{-\alpha}_{1}\phi}_{\alpha_{1}} & {-\alpha}_{1} & \xi_{1} & 0 \\ {-\alpha}_{2} & {-\alpha}_{2} & 0 & \xi_{2} \end{matrix} \right]$$

The next generation matrix is defined as

$$K=FV^{-1}=\left[ \begin{matrix} 0 & 0 & \phi_{\beta_{1}}\beta_{1}(1-\pi) & \beta_{2}(1-\pi) \\ 0 & 0 & \beta_{1}\pi& \beta_{2}\pi\\ 0 & 0 & 0 & 0 \\ 0 & 0 & 0 & 0 \end{matrix} \right]\left[ \begin{matrix} 1/\gamma& 0 & 0 & 0 \\ 0 & 1/\gamma& 0 & 0 \\ {\alpha_{1}\phi}_{\alpha_{1}}/(\gamma\xi_{1}) & \alpha_{1}/(\gamma\xi_{1}) & 1/\xi_{1} & 0 \\ \alpha_{2}/(\gamma\xi_{2}) & \alpha_{2}/(\gamma\xi_{2}) & 0 & 1/\xi_{2} \end{matrix} \right]$$

$$=\left[ \begin{matrix} \frac{\phi_{\beta_{1}}\beta_{1}(1-\pi){\alpha_{1}\phi}_{\alpha_{1}}}{\gamma\xi_{1}}+\frac{\beta_{2}(1-\pi)\alpha_{2}}{\gamma\xi_{2}} & \frac{\phi_{\beta_{1}}\beta_{1}(1-\pi)\alpha_{1}}{\gamma\xi_{1}}+\frac{\beta_{2}(1-\pi)\alpha_{2}}{\gamma\xi_{2}} & \frac{\phi_{\beta_{1}}\beta_{1}(1-\pi)}{\xi_{1}} & \frac{\beta_{2}(1-\pi)}{\xi_{2}} \\ \frac{\beta_{1}\pi{\alpha_{1}\phi}_{\alpha_{1}}}{\gamma\xi_{1}}+\frac{\beta_{2}\pi\alpha_{2}}{\gamma\xi_{2}} & \frac{\beta_{1}\pi\alpha_{1}}{\gamma\xi_{1}}+\frac{\beta_{2}\pi\alpha_{2}}{\gamma\xi_{2}} & \frac{\beta_{1}\pi}{\xi_{1}} & \frac{\beta_{2}\pi}{\xi_{2}} \\ 0 & 0 & 0 & 0 \\ 0 & 0 & 0 & 0 \end{matrix} \right]$$

Using the notation $R_{0,i}=\frac{\alpha_{i}\beta_{i}}{\xi_{i}\gamma}$, we have

$$K= \left[ \begin{matrix} (1-\pi)(\phi_{\beta_{1}}\phi_{\alpha_{1}}R_{0,1}+R_{0,2}) & (1-\pi)(\phi_{\beta_{1}}R_{0,1}+R_{0,2}) & \frac{\phi_{\beta_{1}}\beta_{1}(1-\pi)}{\xi_{1}} & \frac{\beta_{2}(1-\pi)}{\xi_{2}} \\ \pi(\phi_{\alpha_{1}}R_{0,1}+R_{0,2}) & \pi(R_{0,1}+R_{0,2}) & \frac{\beta_{1}\pi}{\xi_{1}} & \frac{\beta_{2}\pi}{\xi_{2}} \\ 0 & 0 & 0 & 0 \\ 0 & 0 & 0 & 0 \end{matrix} \right]$$

The largest eigenvalue of $K$ is $R_{0}^{\phi}.$ The eigenvalues of K are two zeros and the following, where $K_{ij}$ refers to the ijth entry of $K$:

$$\frac{1}{2}\left( K_{11}+K_{22} \right)\pm\frac{1}{2}\sqrt{\left( K_{11}+K_{22} \right)^{2}-4\left( K_{11}K_{22}-K_{12}K_{21} \right)}.$$

We have

$$K_{11}+K_{22}=\left( 1-\pi\right)\left( \phi_{\beta_{1}}\phi_{\alpha_{1}}R_{0,1}+R_{0,2} \right)+ \pi\left( R_{0,1}+R_{0,2} \right)=R_{0,1}\left( \pi+\left( 1-\pi\right)\phi_{\beta_{1}}\phi_{\alpha_{1}} \right)+R_{0,2}$$

and

$$K_{11}K_{22}-K_{12}K_{21}=\left( 1-\pi\right)\pi\left( \phi_{\beta_{1}}\phi_{\alpha_{1}}R_{0,1}+R_{0,2} \right) \left( R_{0,1}+R_{0,2} \right)$$

$$-(1-\pi)\pi(\phi_{\beta_{1}}R_{0,1}+R_{0,2})(\phi_{\alpha_{1}}R_{0,1}+R_{0,2})$$

$$=\left( 1-\pi\right)\pi\left( \phi_{\beta_{1}}\phi_{\alpha_{1}}R_{0,1}R_{0,1}+R_{0,2}R_{0,1}+\phi_{\beta_{1}}\phi_{\alpha_{1}}R_{0,1}R_{0,2}+R_{0,2}R_{0,2} \right)$$

$$-(1-\pi)\pi(\phi_{\beta_{1}}R_{0,1}\phi_{\alpha_{1}}R_{0,1}+R_{0,2}\phi_{\alpha_{1}}R_{0,1}+\phi_{\beta_{1}}R_{0,1}R_{0,2}+R_{0,2}R_{0,2})$$

$$=\left( 1-\pi\right)\pi\left( {R_{0,1}R}_{0,2}+\phi_{\beta_{1}}\phi_{\alpha_{1}}R_{0,1}R_{0,2}-(\phi_{\alpha_{1}}+\phi_{\beta_{1}})R_{0,1}R_{0,2} \right).$$

From these expressions, $R_{0}^{\phi}$ can be calculated explicitly, but it is not necessary for our purposes. Instead, are interested in the expression of $R_{0}$ in the absence of intervention, i.e., with $\phi_{\beta_{1}}=\phi_{\alpha_{1}}=1$. Without intervention, $K_{11}K_{22}-K_{12}K_{21}=0$, and the whole expression simplifies to

$$R_{0}=R_{0,1}+R_{0,2}.$$

If we were to further simplify by removing a transmission pathway (by setting $\beta_{2}=0$ or $\beta_{1}=0),$this expression would simplify to the remaining pathway-specific reproduction number $R_{0,1}$ or $R_{0,2}$, respectively.
